# Exercise therapy for gestational diabetes mellitus: from basic to clinical

**DOI:** 10.3389/fmed.2025.1716883

**Published:** 2025-12-19

**Authors:** Xiaying Niu, Kaiwen Wang, Li Li, Lin Zhang

**Affiliations:** 1 Department of Internal Medicine, Beijing Obstetrics and Gynecology Hospital, Beijing Maternal and Child Health Care Hospital, Capital Medical University, Beijing, China; 2 Department of Hospital Administration Office, Beijing Obstetrics and Gynecology Hospital, Beijing Maternal and Child Health Care Hospital, Capital Medical University, Beijing, China

**Keywords:** aerobic exercise, exercise, gestational diabetes mellitus, glucose metabolism, insulin resistance, strength training

## Abstract

This article reviews exercise therapy for gestational diabetes mellitus (GDM). First, the pathophysiological basis of GDM was described, including epidemiological characteristics, pathological mechanisms, and progress in diagnostic techniques. The basic theory of exercise therapy is discussed, including the effect of exercise on the pathophysiology of GDM, the biological mechanism, and the comparison of the curative effect of different forms of exercise. This was followed by an in-depth analysis of clinical exercise therapy practices including exercise prescription formulation, clinical trial analysis, and safety assessment. Simultaneously, the technical progress of exercise therapy is introduced, including the application of exercise monitoring technology and digital health tools, as well as the development and application of personalized exercise therapy programs. In addition, the controversies and challenges of exercise therapy, such as indications and contraindications, compliance issues, and long-term efficacy evaluations, are discussed. Finally, the research trend, the potential of innovative exercise therapy, and the prospect of multidisciplinary cooperation are discussed to provide a comprehensive and in-depth theoretical and practical reference for exercise therapy for GDM.

## Introduction

Gestational diabetes mellitus (GDM) is a glucose intolerance disease that first appears during pregnancy and seriously affects the health of mothers and infants. Although its global prevalence varies widely due to differences in diagnostic criteria, population characteristics, and lifestyle factors, most regions report a substantial and increasing disease burden (1). Established maternal risk factors include older age, elevated pre-pregnancy body mass index (BMI), a family history of diabetes, previous GDM, and excessive gestational weight gain (2–4).

The pathophysiology of GDM is multifactorial. Insulin resistance—exacerbated by placental hormones such as human placental lactogen, estrogen, and progesterone—and inadequate β-cell compensation represent central mechanisms (5). In addition, inflammation, oxidative stress, and alterations in gut microbiota composition contribute to impaired glucose metabolism and dysregulated immune responses in GDM (6, 7). Despite the essential role of the oral glucose tolerance test (OGTT) in diagnosis, thresholds vary across international guidelines, and attempts at earlier screening or metabolomics-based biomarker identification require further validation (8, 9).

Given these metabolic disturbances, exercise therapy has emerged as a key non-pharmacological strategy for preventing and managing GDM (10). Regular physical activity improves glucose uptake, enhances insulin sensitivity, modulates inflammatory and lipid profiles, and helps prevent excessive gestational weight gain (11). Multiple exercise modalities—including aerobic, resistance, and combined training—have been investigated, and accumulating evidence suggests that structured exercise programs can reduce GDM incidence and improve metabolic outcomes (10–12). However, previous reviews have often synthesized older studies conducted under outdated diagnostic or exercise criteria (13), highlighting the need for an updated overview based on recent literature.

Therefore, this review synthesizes evidence from SCIE-indexed publications within the past decade to summarize the mechanisms, efficacy, and clinical feasibility of exercise interventions in GDM, with the aim of providing clearer guidance for developing effective and practical exercise strategies for pregnant women with GDM.

## Epidemiological study of gestational diabetes mellitus

### Global prevalence of gestational diabetes mellitus

In recent years, with the increasing incidence of diabetes worldwide, the prevalence of GDM has attracted considerable attention. Studies have shown that Vitamin D deficiency is prevalent worldwide and is associated with an increased risk of various human diseases. Vitamin D deficiency is particularly common in pregnant women and is associated with adverse pregnancy outcomes such as GDM (14). Although diabetes affects numerous populations at different life stages, the global burden of GDM has not been fully assessed, especially in developing countries, and there is a lack of systematically integrated data to estimate its global prevalence (15).

The prevalence of GDM varies widely worldwide, ranging from 2 to 18% in different regions and countries, which is mainly attributed to the heterogeneity of screening methods, diagnostic criteria, and population characteristics (16). For example, the global prevalence of GDM is estimated to be 16.2%, as extrapolated from the International Diabetes Federation, but the actual reported prevalence may be underestimated due to factors such as access to resources and attitudes toward screening, particularly among foreign-born females and indigenous populations (17). In addition, the global rise in obesity makes more women of childbearing age overweight or obese, which increases the risk of GDM (18). In some regions, such as the Mexican border, the morbidity risk of GDM is significantly increased owing to the high prevalence of overweight, obesity, and family history of type 2 diabetes in women (19).

### Incidence of gestational diabetes mellitus in different populations

There were significant differences in the incidence of GDM among different populations. In a Spanish study, 5,087 pregnant women were analyzed and it was found that primiparas reported better self-perceived health and nutritional balance and a lower incidence of GDM. On the other hand, multiparous women showed healthier lifestyle habits but had a relatively higher incidence of GDM (20). In twin pregnancies, the prevalence of glucose intolerance was 17.5%, but glucose intolerance in twin pregnancies was not associated with an increased risk of macrosomia or cesarean delivery (21).

Racial differences also have an impact on GDM incidence. For example, South Asian women have relatively high levels of subcutaneous fat and serum leptin in the first trimester of pregnancy, which increases their risk of GDM and retains more weight and subcutaneous fat after delivery, further increasing the risk of GDM in future pregnancies (22). In a prospective study in Tianjin, China, the adjusted prevalence of GDM was 8.1% using the 1999 World Health Organization (WHO) criteria and further increased to 9.3% using the International Diabetes and Pregnancy Study Group (IADPSG) criteria. Advanced age, higher pre-pregnancy body mass index, Han nationality, higher systolic blood pressure, family history of diabetes, weight gain during pregnancy, and habitual smoking are risk factors for GDM (23). In sub-Saharan Africa, the prevalence of GDM varies widely from study to study, ranging from 3.2 to 5.1% when different diagnostic criteria are used, with overweight and/or obesity, family history of type 2 diabetes, previous stillbirth, previous macrosomia delivery, and age > 30 years being common risk factors (24).

### Analysis of risk factors of gestational diabetes mellitus

The occurrence of GDM is affected by several factors. In an Australian cohort study of female reproductive characteristics and chronic disease risk, approximately 10% of participants reported a history of GDM (25). According to a prospective study from the UK Biobank, women with a history of GDM have an increased risk of cardiovascular disease, including coronary artery disease, myocardial infarction, and ischemic stroke, and traditional risk factors such as diabetes, hypertension, and dyslipidemia partially mediate this relationship (26), according to a prospective study from the UK Biobank.

**Figure 1:**
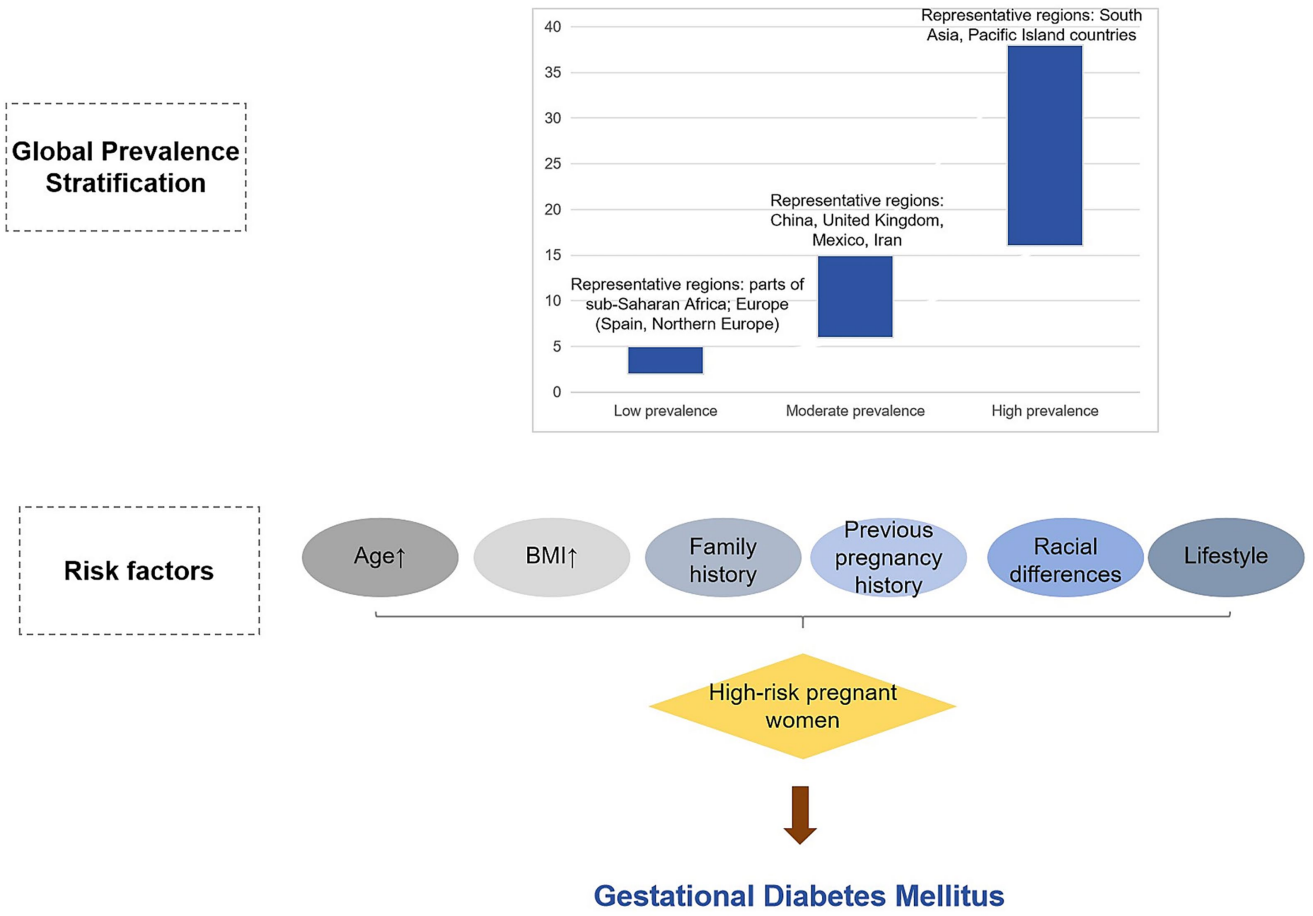
Stratification of global prevalence of gestational diabetes mellitus (GDM). This figure illustrates the global stratification of gestational diabetes mellitus (GDM) prevalence. Regions are classified based on reported prevalence rates, with low (2–5%), moderate (6–15%), and high (16–38%) prevalence levels. The figure highlights geographic variations, with high prevalence found in South Asia and Pacific Island countries, and lower prevalence in parts of sub-Saharan Africa and Northern Europe.

A retrospective analysis of 1,598 pregnant women in China found that GDM was an independent risk factor for premature delivery (< 32 gestational weeks) and premature delivery (32–37 gestational weeks) (27). In a study of a cohort of very early preterm infants, increased maternal pre-pregnancy body mass index and diabetes were found to be associated with an increased risk of attention-deficit hyperactivity disorder (ADHD) at 10 and 15 years of age, and there was a positive interaction between maternal pre-pregnancy body mass index and diabetes (28). In addition, lifestyle factors are associated with the risk of GDM. For example, in a study of rural areas in Telangana, India, it was found that more women with GDM were identified according to the WHO criteria than the DIPSI criteria, while the DIPSI criteria may miss many cases. Pregnant women are at an increased risk of future diabetes (29) (Figure 1).

## Diagnostic techniques for gestational diabetes mellitus

### Evolution of screening criteria for gestational diabetes mellitus

Screening criteria for GDM have evolved in several stages. Previously, there were differences in the screening criteria adopted by different regions and institutions, which led to inconsistencies in the diagnosis of GDM. For example, Cuba screens all pregnant women with a fasting plasma glucose test and uses the modified WHO criteria for diagnosis, while some international recommendations skip the screening step and directly follow the diagnostic criteria of the Hyperglycemia and Adverse Pregnancy Outcomes Study (16).

With a deepening understanding of GDM, there are some trends of unification in the world. In 2010, the International Diabetes and Pregnancy Study Group (IADPSG) proposed a new diagnostic criterion that uses a 75 g oral glucose tolerance test (OGTT) to diagnose GDM as long as one blood glucose value exceeds the threshold, which has increased the diagnostic rate of GDM (30). In an Australian study comparing the impact of traditional screening methods with IADPSG guidelines on pregnancy outcomes, it was found that the adoption of IADPSG guidelines increased the diagnosis of GDM but did not reduce adverse pregnancy outcomes (31). In addition, during the COVID-19 pandemic, some temporary GDM screening criteria were recommended, such as in the Australian study, which found that 25.3% of GDM patients were missed using these temporary criteria, highlighting the priority of OGTT testing in high-risk patients (32).

### Application of oral glucose tolerance test

The oral glucose tolerance test (OGTT) remains the cornerstone and most widely accepted method for diagnosing gestational diabetes mellitus, as it provides a direct assessment of maternal glucose tolerance during pregnancy. For example, in a study of 95 participants treated with *Momordica charantia* and glibenclamide, OGTT assessment found that the glibenclamide group had a significant improvement in the 2-h OGTT results, while the *Momordica charantia* group, although less hypoglycemic, was more effective in improving diabetes-related cardiovascular risk factors (33).

Most high-quality guidelines recommend the one-step 75 g OGTT between 24 and 28 weeks of gestation, and variations in diagnostic thresholds across international organizations contribute substantially to differences in reported GDM prevalence (34). Several recent studies have evaluated whether early pregnancy OGTT or modified cut-off values can improve early detection; however, early testing has been shown to produce a high rate of false-positive diagnoses and limited predictive value for GDM diagnosed later in pregnancy (35, 36). Collectively, these findings reinforce that despite ongoing discussion regarding optimization and early assessment, the OGTT remains the most reliable and clinically validated diagnostic tool for identifying GDM.

### Application of new diagnostic techniques in gestational diabetes mellitus

Emerging diagnostic techniques provide a new method for the early diagnosis of GDM. Circulating microRNAs (miRNAs) have been identified as potential biomarkers for the early diagnosis of GDM. In one study, miR-223 and miR-23a were found to be upregulated in GDM patients through the detection of miRNAs in the plasma of pregnant women, and a logistic regression model using these two miRNAs achieved an AUC value of 0.91 with high diagnostic accuracy (37).

Additionally, some studies have focused on the relationship between specific gene variants and GDM. For example, the interaction of the rs7754840 variant of CDKAL1 with lifestyle intervention on changes in glycemic markers was studied in women with a history of GDM, and women carrying the C allele of this variant had increased fasting insulin and HOMA-IR in the control group and the opposite in the intervention group. This suggests that healthy lifestyle interventions may be beneficial in improving insulin resistance in women with this genetic variant (38). At the same time, studies on placental tissues of GDM patients have found that changes in the expression of some genes may be related to the occurrence and development of GDM, which provides a new potential target (39) for the diagnosis and treatment of GDM.

Beyond circulating biomarkers and genomic indicators, advanced imaging technologies have recently emerged as valuable complementary tools for assessing maternal health in GDM. Speckle-tracking echocardiography (STE), an imaging modality capable of quantifying myocardial deformation with high sensitivity, can detect subtle ventricular dysfunction even when conventional echocardiographic measurements appear normal (40, 41). Recent evidence suggests that women with GDM may exhibit reductions in global longitudinal strain and other early changes in myocardial mechanics, indicating the presence of subclinical myocardial impairment (42). Incorporating STE into the diagnostic framework may therefore enhance cardiovascular risk assessment and support earlier monitoring or intervention in this high-risk population.

## Genetic basis of gestational diabetes mellitus

### Genetic susceptibility of gestational diabetes mellitus

Several studies have shown that genetic factors play an important role in the development of GDM. In a South Indian population study, 518 women with GDM and 910 pregnant women with normal glucose tolerance were genotyped, and rs7754840 and rs7756992 single nucleotide polymorphisms (SNPs) in the CDK5 regulatory subunit-associated protein 1-like 1 (CDKAL1) gene were found to be significantly associated with GDM. Their minor alleles showed susceptibility to GDM with odds ratios of 1.34 and 1.45, respectively (43).

In another study, 240 pregnant women with GDM and 330 healthy pregnant women were analyzed, and it was found that the macrophage migration inhibitory factor (MIF) rs1007888 single nucleotide polymorphism was associated with genetic susceptibility to GDM. GDM patients with the GG genotype have higher levels of fasting blood glucose, fasting insulin, and HOMA-insulin resistance. However, the HOMA-b value was lower, suggesting that the GG genotype may be a genetic susceptibility factor in the morbidity mechanism of GDM (44). In addition, maternal hyperglycemia was found to be related to the epigenetic regulation of the neonatal leptin gene by Mendelian randomization analysis, which supported that maternal glucose was one of the causal pathways affecting the epigenetic regulation of leptin in offspring (45).

### Identification and functional analysis of related genes

Identification and functional analysis of GDM-related genes will help us to understand the mechanism of GDM morbidity. In the aspect of gene identification, many genes were found to be related to the occurrence of GDM through gene research in different populations. For example, in a study of Chinese women, some genetic variants were found to be associated with the risk of GDM, such as the rs7903146 polymorphism of the TCF7L2 gene, which was associated with GDM susceptibility in the overall population as well as in white, Hispanic/Latino, and Asian subgroups, with Asians having the highest risk of carrying the TT homozygous allele (46).

Functional analysis revealed that some genes were involved in GDM morbidity of GDM by affecting insulin secretion and metabolic regulation. For example, the rs10830963 polymorphism in the MTNR1B gene interacts with lifestyle interventions for GDM, and in high-risk women, those who do not carry the risk allele G benefit more from lifestyle interventions, suggesting that some genetic risk variants may alter the effectiveness of lifestyle interventions (47). In addition, studies of fetal imprinted genes have found that polymorphic variants of fetal imprinted genes, particularly in the IGF2/INS region, contribute to the risk of maternal glucose concentration in the third trimester (48).

### Familial aggregation of gestational diabetes mellitus

A certain degree of familial aggregation was observed in GDM. In a study on childhood diabetes in Taiwan, maternal GDM was found to be a risk factor for type 1 diabetes in children, with an odds ratio of 4.36 (49). In a study of Iranian women, 110 women with a history of GDM were followed up 1–6 years postpartum and it was found that 32.7% of women developed type 2 diabetes and that women with a family history of diabetes had a higher risk of developing GDM (50).

In a study of pregnant women in Tianjin, China, it was found that a family history of diabetes was one of the risk factors for GDM, and the impact of family history on the risk of GDM morbidity was different among different blood groups. For example, the risk of GDM in women in the non-AB blood group was higher than that in the AB blood group, and this difference was more obvious in women with a family history of diabetes (51). In addition, in a systematic review of GDM in sub-Saharan Africa, a family history of type 2 diabetes was found to be an important risk factor for GDM (24).

## Pathophysiological mechanism of gestational diabetes mellitus

### The role of insulin resistance in gestational diabetes mellitus

Insulin resistance is a key factor in the morbidity of GDM. Studies have shown that GDM patients have significant insulin resistance, which is manifested by increased fasting blood glucose, fasting insulin, and HOMA-insulin resistance and decreased HOMA-b levels (44). In a GDM rat model, obvious pathological changes were observed in the livers of offspring rats, such as hepatocyte edema, increased apoptosis, increased autophagosomes, and changes in the expression of related proteins, suggesting that insulin resistance may lead to the development of GDM by affecting liver metabolism (52).

In addition, some studies have focused on the relationship between insulin resistance and other factors. For example, in a study of GDM patients, some inflammatory factors and oxidative stress indicators in serum were found to be associated with insulin resistance, such as increased levels of serum pentraxin-3 (PTX3) and high-sensitivity C-reactive protein (hs-CRP) in GDM patients. PTX3 was positively correlated with hs-CRP, body mass index, fasting blood glucose, and HOMA-IR, suggesting that inflammation and oxidative stress may be involved in the development of insulin resistance in GDM (53). At the same time, in the nutritional intervention study of GDM patients, it was found that probiotics may have beneficial effects on GDM by improving insulin resistance, such as reducing HOMA-IR and increasing QUICKI (54).

### Effect of placental hormones on glucose metabolism

Placental hormones play an important role in regulating maternal glucose metabolism during pregnancy. During normal pregnancy, a variety of hormones secreted by the placenta, such as human placental lactogen, estrogen, and progesterone, lead to a gradual increase in maternal insulin resistance to meet the energy needs of fetal growth and development (55). However, GDM may occur when the mother is unable to compensate for insulin resistance.

It has been found that the secretion and function of placental hormones may be abnormal in patients with GDM. For example, in a study on placental tissue in patients with GDM, it was found that the expression of some genes related to hormone synthesis and metabolism was altered, which may affect the normal function of placental hormones (56). In addition, studies have shown that abnormalities in placental hormones may lead to disorders of maternal hepatic glucose metabolism by affecting the activity and expression of enzymes related to glucose metabolism in the liver, thereby affecting blood glucose levels (57). At the same time, in a study of twin pregnancy with GDM, it was found that twin pregnancy may be diagnosed with GDM earlier, and insulin therapy may be started earlier, suggesting that the effect of placental hormones on glucose metabolism may be more significant in twin pregnancy (58).

### Metabolic pathway of gestational diabetes mellitus

Multiple metabolic pathways exist in GDM. In a study on circulating retinol-binding protein 4 (RBP4) levels in GDM patients, a meta-analysis showed that circulating RBP4 levels in Asian women with GDM in the second and third trimesters of pregnancy were significantly higher than those in normal controls, suggesting that RBP4 may be involved in the metabolic abnormalities of GDM (59).

**Figure 2:**
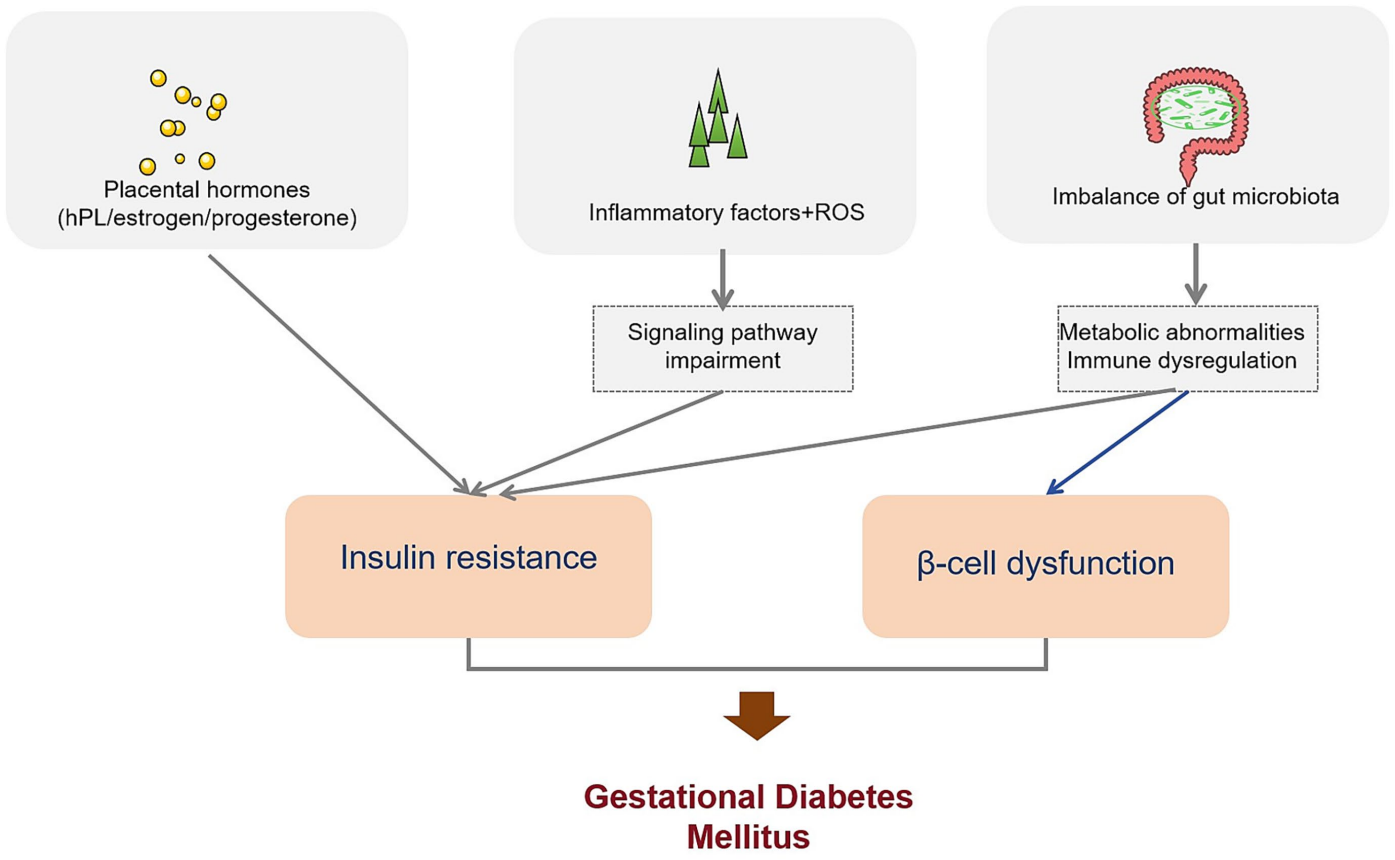
The pathogenesis of gestational diabetes mellitus. This figure depicts the pathophysiological mechanisms underlying gestational diabetes mellitus (GDM). It shows how placental hormones, inflammatory factors, and gut microbiota imbalances contribute to insulin resistance and β-cell dysfunction. These factors interact to impair insulin signaling, ultimately resulting in GDM.

In addition, the study also found that GDM patients have lipid metabolism disorders. In a study of GDM patients, it was found that the levels of blood lipid indicators, such as triglyceride and total cholesterol, were elevated and related to insulin resistance and inflammatory response (60). At the same time, the gut microbiota may also play a role in the metabolic abnormalities of GDM. The study of metabolites derived from gut microbiota in GDM patients found that the levels of some metabolites such as short-chain fatty acids and bile acids were altered and were related to blood glucose and lipid levels, suggesting that metabolic disorders of gut microbiota may be involved in the morbidity mechanism of GDM (61). In addition, in a GDM mouse model, maternal hyperglycemia was found to affect the lipid metabolism of offspring and susceptibility to diet-induced obesity, further indicating that GDM has an abnormal metabolic pathway affecting the health of offspring (62) (Figure 2).

## Basic theory of exercise therapy for gestational diabetes mellitus

### Effect of exercise on pathophysiology of gestational diabetes mellitus

Exercise has multiple effects on GDM pathophysiology of GDM. From the perspective of energy metabolism, exercise can increase muscle glucose uptake and utilization, improve insulin sensitivity, and help regulate blood sugar levels. Studies have shown that regular exercise can reduce the risk of morbidity in GDM, and exercise can help control blood sugar levels in patients with GDM (63).

In a study of 253 pregnant women, poor dietary patterns, such as highly refined grains, fats, oils, and fruit juices, as well as intake patterns high in added sugars and organ meats and low in fruits and vegetables, were associated with an increased risk of GDM, and appropriate exercise combined with a healthy diet may improve this condition (64). Exercise may also affect fat metabolism and the levels of inflammatory cytokines. Studies have pointed out that exercise during pregnancy may have a positive effect on reducing triglyceride, TNF-*α* and leptin levels as well as increasing muscle-derived IL-6 levels. Although the evidence base is small, it suggests the potential benefits of exercise on metabolic regulation in GDM patients (65). In addition, exercise may affect the pathophysiological process of GDM by regulating the signaling pathways related to placental function and fetal growth and development; however, more research is needed to clarify the specific mechanism (66).

Beyond its well-established metabolic benefits, exercise may also play a role in improving or preventing subclinical cardiac alterations in women with GDM. Recent evidence indicates that GDM is associated with subtle impairments in myocardial mechanics—such as reduced global longitudinal strain—detectable through STE, even when conventional echocardiographic parameters appear normal. Moderate-intensity physical activity has been shown in pregnant women with cardiometabolic risk factors to improve endothelial function, reduce systemic inflammation, and attenuate hemodynamic stress, all of which may contribute to preserving myocardial performance. Although direct data specifically in GDM remain limited, the mechanistic pathways through which exercise improves insulin sensitivity, reduces oxidative stress, and modulates adipokine profiles suggest a potential protective effect on maternal cardiac function. Future research integrating structured exercise interventions with STE-based cardiac monitoring may further clarify this relationship.

### Biological mechanism of exercise therapy for gestational diabetes mellitus

There are many biological mechanisms underlying the therapeutic effects of exercise in GDM. On the one hand, exercise can regulate insulin signaling pathways. In animal experiments, exercise increased the phosphorylation of insulin receptor substrate-1 (IRS-1) and enhanced insulin signaling, thus promoting the translocation of glucose transporter 4 (GLUT4) to the cell membrane, increasing the uptake of glucose by cells, and improving insulin resistance (67).

In contrast, exercise may also affect adipokine secretion. Irisin, an exercise-induced myokine, increases energy expenditure and improves carbohydrate homeostasis. Studies have found that irisin levels may be altered in patients with GDM, affecting fat metabolism and insulin sensitivity (68). Exercise may also affect the production of short-chain fatty acids and other metabolites by regulating intestinal flora, thereby affecting the occurrence and development of GDM. The intestinal flora is closely related to host metabolism. Exercise may provide a new method for the treatment of GDM by improving the composition and function of intestinal flora, alleviating the inflammatory response, and improving insulin sensitivity (66).

### Effect of different exercise on gestational diabetes mellitus

There are some differences in the efficacy of the different forms of exercise in GDM. Aerobic exercises, such as walking, swimming, and cycling, have been widely studied for the prevention and treatment of GDM. A systematic review and meta-analysis showed that aerobic exercise during pregnancy reduced the risk of GDM by 28% (69).

**Figure 3:**
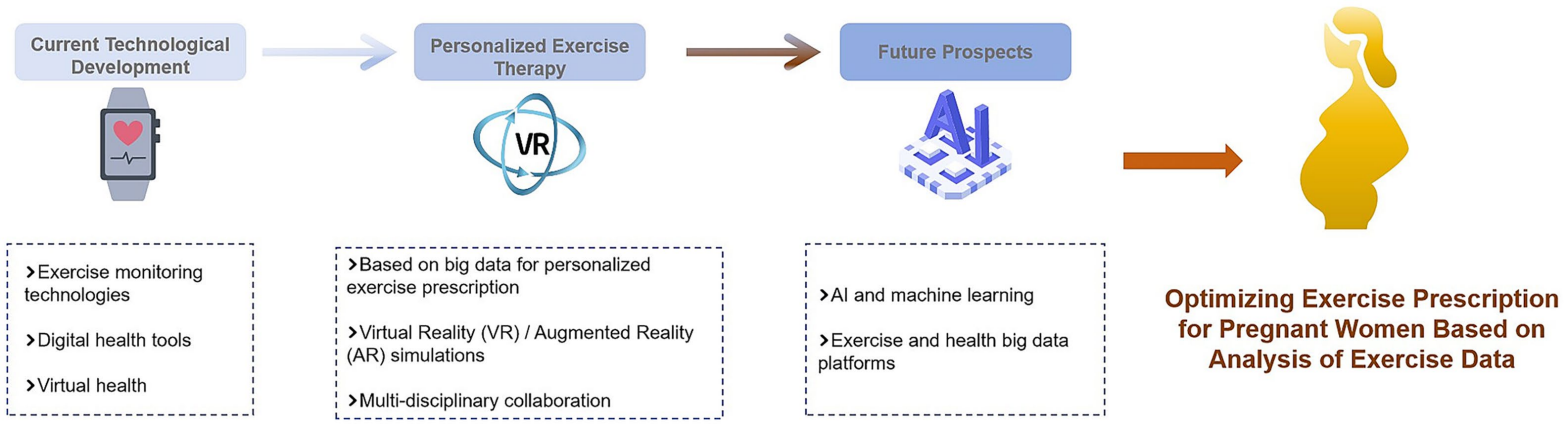
The mechanism of exercise therapy. This figure illustrates how exercise interventions improve metabolic control in pregnant women with gestational diabetes mellitus (GDM). It highlights the positive effects of exercise on skeletal muscle glucose uptake, adipose tissue metabolism, inflammation reduction, and placental function. These mechanisms work together to improve insulin sensitivity and blood glucose regulation.

Resistance training is an important type of exercise. Some studies have compared the effects of aerobic exercise, resistance training, and their combination on GDM patients, and found that different exercise modes have their own characteristics in improving blood sugar control and insulin sensitivity. For example, resistance training may be more helpful in increasing muscle mass and basal metabolic rate, which indirectly improves blood glucose levels (70). Water exercise, such as water aerobic exercise, exerts less pressure on pregnant women’s joints and is also a concern. Studies have compared land exercise, water exercise, and their combination and found that combined exercise or water exercise may be more effective in preventing GDM, while land exercise is more effective in preventing excessive weight gain in pregnant women (71). However, there are some differences in the results of different studies, which may be related to the design of the exercise programs, the characteristics of the subjects, and other factors (Figure 3).

## Clinical exercise therapy for gestational diabetes mellitus

### Exercise prescription in gestational diabetes mellitus

Many factors should be considered when formulating exercise prescriptions for patients with GDM. In terms of exercise type, it is recommended to combine aerobic and resistance exercises. Aerobic exercises, such as walking, swimming, and cycling, can improve cardiopulmonary function and enhance metabolic capacity in the body. Resistance exercises, such as the use of elastic bands and simple equipment training, help increase muscle mass and basal metabolic rate (72).

Moderate-intensity exercise is generally recommended, typically corresponding to 50–70% of age-predicted maximal heart rate; for example, an appropriate training heart rate for a 30-year-old pregnant woman would be approximately 95–133 beats per minute. Research showing the greatest reduction in GDM risk typically prescribe at least 150 min of moderate-intensity exercise per week, delivered as ≥3 sessions per week with 30–60 min per session (72). Importantly, variations in exercise dose and adherence across trials have contributed to differences in reported effectiveness, highlighting the need for adequate intensity and consistent participation to achieve meaningful metabolic improvements.

Exercise sessions should be distributed throughout the week to avoid excessive fatigue. Warm-up activities, such as slow walking and joint mobilization, are recommended before exercise, while stretching and relaxation should be performed afterward to reduce muscle soreness and prevent injury. For pregnant women with obesity, comorbidities, or limited physical capacity, exercise prescriptions should be individualized and ideally supervised by trained professionals to ensure safety and adherence (73).

Overall, current evidence suggests that moderate-intensity aerobic or combined aerobic–resistance exercise totaling approximately 150 min per week yields the most consistent improvements in glycemic control, insulin sensitivity, gestational weight gain, and overall metabolic outcomes in women with GDM, making these modalities the most supported exercise options in clinical practice.

### Clinical trial analysis of exercise therapy for gestational diabetes mellitus

Several clinical trials have explored the effects of exercise therapy on GDM, but the findings have not been entirely consistent. In a randomized controlled trial, a 12-week individualized, motivation-matched exercise intervention for pregnant women at risk of GDM yielded a relative odds ratio of 0.61 for GDM in the exercise group; however, the difference did not reach statistical significance, and no improvements were observed in birth outcomes (74). The limited effect in this trial may be attributable to modest exercise intensity, variable adherence, or initiation of the intervention later in pregnancy—factors that often attenuate intervention efficacy.

In contrast, another study involving overweight or obese pregnant women introduced supervised exercise early in pregnancy and found a significantly lower incidence of GDM in the intervention group, along with reduced gestational weight gain at 25 weeks and at term and decreased insulin resistance (75). The stronger outcomes in this trial may reflect earlier initiation, higher exercise dosage, and more consistent participant adherence. Additional studies examining inflammatory markers, such as C-reactive protein (CRP), have reported a downward trend following exercise intervention; however, the differences between the intervention and comparison groups did not reach statistical significance (76), possibly due to short intervention duration or insufficient exercise intensity to meaningfully alter systemic inflammation.

Taken together, these trials illustrate both the potential benefits and the variability of exercise therapy in GDM. Differences in study design, sample size, baseline risk profiles, intervention timing, exercise type, and adherence likely contribute to inconsistent findings across studies. Trials with higher methodological rigor, standardized exercise protocols, and adequate supervision tend to report more favorable metabolic outcomes. Therefore, while current evidence supports the therapeutic value of exercise, additional large-scale, well-controlled studies are needed to clarify the optimal exercise dose, timing, and modality for GDM prevention and management.

### Safety evaluation of exercise therapy for gestational diabetes mellitus

The safety of exercise therapy for GDM is a critical consideration in clinical practice. Most studies have shown that moderate exercise is safe for pregnant women and fetuses in the absence of contraindications. A systematic review and meta-analysis involving normal-weight pregnant women demonstrated that aerobic exercise performed 3–4 times per week for 35–90 min was not associated with an increased risk of preterm birth. Moreover, the exercise group showed a lower cesarean section rate and significantly reduced incidences of GDM and hypertensive disorders compared with the control group (77). These findings support the safety and potential obstetric benefits of structured prenatal exercise.

However, safety outcomes across studies are not entirely uniform and should be interpreted in light of methodological differences. Many trials rely on self-reported adherence or lack continuous monitoring, which may underestimate potential discomfort or early signs of intolerance. In addition, populations with obesity, multiple comorbidities, or high obstetric risk are underrepresented in some studies, limiting the generalizability of safety conclusions. Exercising under inadequate supervision or at intensities exceeding recommended thresholds may also increase maternal fatigue or orthostatic symptoms, underscoring the importance of adhering to standardized, moderate-intensity protocols.

**Figure 4:**
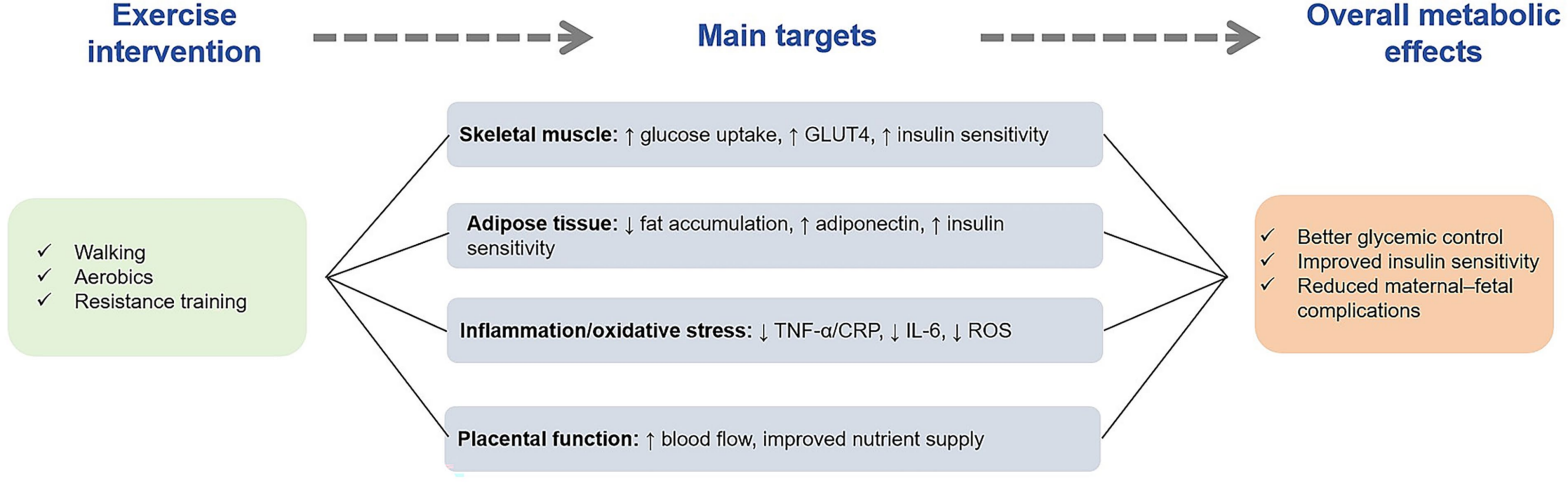
Exercise prescription for gestational diabetes and its clinical application. This figure outlines the key elements of an exercise prescription for managing gestational diabetes mellitus (GDM). It includes recommendations on exercise types (aerobic exercise, resistance training, stretching), frequency, duration, and intensity. Additionally, the figure summarizes the clinical benefits of exercise, such as improved blood glucose control, reduced insulin resistance, and decreased maternal and fetal complications.

Certain pregnant women may have exercise contraindications. Absolute contraindications include placenta previa, vaginal bleeding, and cervical incompetence/cerclage. Relative contraindications, such as chronic hypertension and uncontrolled thyroid disease Pregnant women should fully communicate with obstetricians before exercise to assess the advantages and disadvantages of exercise (78). During exercise, careful monitoring of maternal heart rate, blood pressure, blood glucose, fetal heart rate, and fetal movement is essential, and any discomfort should prompt immediate cessation of activity and medical evaluation. When performed within established safety parameters and under appropriate supervision, exercise therapy remains a safe and feasible intervention for women with GDM (Figure 4).

## Technical progress of exercise therapy for gestational diabetes mellitus

### Application of exercise monitoring technology in gestational diabetes mellitus

Exercise monitoring technology provides strong support for GDM management of GDM. Wearable devices such as Fitbit and other activity trackers can objectively record pregnant women’s exercise steps, exercise time, exercise intensity, and other information, help pregnant women better understand their exercise situation, and provide a basis for doctors to adjust exercise prescriptions (79, 80).

In one study, pregnant women wore activity trackers to record daily exercise data from recruitment to 20 weeks of gestation and found that objective measures could more accurately assess the level of exercise in pregnant women and its relationship with pregnancy outcomes, such as GDM (79). In addition, in combination with mobile applications, real-time synchronization and analysis of motion data can be achieved. For example, the eMOM mobile application, which not only connects to continuous glucose monitors and activity trackers but also includes a food diary function, improves self-management of pregnant women in terms of diet and exercise, optimizes fasting blood sugar, weight gain, and other indicators, and reduces the incidence of neonatal macrosomia (81). The application of these techniques can help improve the effects of exercise therapy and compliance of pregnant women.

### Digital health tools assist exercise therapy for gestational diabetes mellitus

Digital health tools are playing an increasingly important role in exercise therapy for gestational diabetes. Smartphone applications (APP) can provide personalized exercise and diet advice, popularization of health knowledge, blood sugar monitoring records, and other functions. For example, Pregnant + APP can automatically transmit blood sugar levels and provide healthy diet and exercise information to help pregnant women better manage GDM (82). Some APPs also combine behavior-change technologies, such as self-monitoring, goal setting, and reminders, to improve the self-management ability of pregnant women. In a study of Hispanic women, the use of educational audiovisual modules, personal action plans, motivational text messaging, and other features significantly improved participants’ healthy eating and physical activity self-efficacy over an 8-week trial (83). Telemedicine extends these benefits by enabling remote consultations and monitoring, allowing clinicians to review patients’ exercise behaviors, glycemic trends, and pregnancy status in real time, thereby increasing the convenience and responsiveness of clinical care (84).

Despite these advantages, several barriers may limit the widespread implementation of digital health in GDM management. Variability in digital literacy, limited access to smartphones or stable internet connections, concerns about data privacy, and inconsistent patient engagement can all reduce the effectiveness of such tools. Additionally, differences in healthcare system integration and the lack of standardized digital platforms may affect clinical uptake. Addressing these barriers is essential for maximizing the potential of digital health technologies to support exercise adherence and improve metabolic outcomes in women with GDM.

### Development and application of individualized exercise therapy program

Individualized exercise therapy is the future development direction of exercise therapy for GDM. By evaluating the physical condition, exercise ability, living habits, and other factors of pregnant women and formulating targeted exercise programs, the effect and safety of exercise therapy can be improved. For example, for obese pregnant women with GDM, exercise programs may focus more on aerobic exercise combined with appropriate resistance training to help control weight and blood sugar levels. For pregnant women with other complications, the exercise regimen should be carefully (73).

Advanced technical methods can be used to develop personalized sports treatment programs. For example, data collected by wearable devices and mobile applications can be used to analyze the movement patterns and body reactions of pregnant women, providing a basis for program adjustment. Simultaneously, combined with artificial intelligence and big data technology, a large number of GDM patients and exercise treatment data can be analyzed, and the best exercise program for patients with different characteristics can be summarized to achieve more accurate personalized treatment. Although the application of personalized exercise therapy in GDM is still in the developmental stage, it has shown good prospects and is expected to provide better treatment services for patients (85).

## Controversies and challenges of exercise therapy for gestational diabetes mellitus

### Indication and contraindication of exercise therapy for gestational diabetes mellitus

Exercise therapy for GDM has a wide range of indications, and exercise is beneficial to most pregnant women with GDM without serious complications. Proper exercise can help control blood sugar, reduce excessive weight gain, and reduce the cesarean section rate (77). However, it is equally important to identify contraindications to exercise therapy. Absolute contraindications include placenta previa, vaginal bleeding, premature membrane rupture, cervical incompetence, and multiple pregnancies with a risk of premature delivery. In these cases, pregnant women should avoid strenuous exercise and only engage in daily activities (78).

Relative contraindications include chronic hypertension, uncontrolled thyroid disease, type 1 diabetes mellitus with poor blood sugar control, and severe anemia. Pregnant women should exercise cautiously under these conditions, and should be closely monitored and guided by doctors. For example, for pregnant women with GDM who have chronic hypertension, exercise may increase the risk of blood pressure fluctuations; therefore, exercise intensity and duration need to be more strictly controlled and blood pressure changes need to be closely monitored. Accurately understanding the indications and contraindications of exercise therapy is key to ensuring the safety and effectiveness of exercise. Doctors need to make a comprehensive assessment and individualized decision making according to the specific conditions of pregnant women (78).

### The compliance of exercise therapy in gestational diabetes mellitus

Compliance with exercise therapy for GDM is an important factor that affects the therapeutic effects. Several studies have shown that the compliance of pregnant women with exercise therapy is generally low. In a study of pregnant women at risk of GDM, although the majority of pregnant women agreed on the importance of exercise to control the disease, actual adherence to regular exercise was low (86).

There are many reasons for the low compliance. From the perspective of pregnant women, physical discomfort, fatigue, and a lack of exercise awareness during pregnancy are common factors. For example, with the progress of pregnancy, pregnant women may have backache, lower limb edema, and other conditions, affecting their enthusiasm for exercise. Some pregnant women do not know enough about exercise therapy and think that diet control is sufficient, ignoring the importance of exercise. From the perspective of external factors, lack of professional guidance, inconvenience of sports facilities, and insufficient family support also reduce the compliance of pregnant women. Some pregnant women do not know how to choose the right way and intensity of exercise, and community or medical institutions provide limited exercise guidance and facilities. In addition, the family’s lack of understanding or support for the movement of pregnant women is also an obstacle. To improve the compliance of pregnant women to exercise therapy, we need to strengthen health education, provide professional guidance, improve the exercise environment, and enhance family support (87).

### Evaluation of long-term effects of exercise therapy on gestational diabetes mellitus

Relatively few studies have evaluated the long-term effects of exercise therapy in GDM. Some studies have focused on the effects of exercise on the postpartum health of pregnant women and long-term health of their offspring. Follow-up of women with a history of GDM revealed that exercise intervention during pregnancy may have some benefits for postpartum weight management. For example, in one study, women in the intervention group retained less weight 12 months after delivery than those in the control group, but the difference was not statistically significant (88).

Studies have shown that maternal exercise during pregnancy may be associated with a lower risk of macrosomia and large-for-gestational-age infants in offspring, but more long-term follow-up studies are needed to clarify the impact on the long-term metabolic health and neurodevelopment of offspring (89). In addition, the effect of exercise therapy on the long-term development of type 2 diabetes in GDM patients is unclear. Although some studies suggest that exercise may reduce the risk of morbidity, evidence is insufficient. Overall, more high-quality studies with long-term follow-up are needed to comprehensively evaluate the long-term effects of exercise therapy in GDM and to provide a stronger basis for clinical practice.

## Future prospects of exercise therapy for gestational diabetes mellitus

### Research trend of exercise therapy for gestational diabetes mellitus

In the future, research on exercise therapy for GDM will show various trends. On the one hand, more attention should be paid to precision and individualization. With the development of gene detection, metabolomics, and other technologies, a more targeted exercise treatment program is expected to be developed through in-depth analysis of the genetic characteristics and metabolic status of pregnant women. For example, by detecting gene polymorphisms related to insulin resistance and fat metabolism, personalized exercise advice can be provided to pregnant women with different GDM (90).

However, research will focus on the combination of exercise therapy and other treatment methods. For example, exercise should be combined with dietary intervention and drug treatment to explore the best comprehensive treatment mode to improve the therapeutic effect. At the same time, strengthening research on the long-term effects of exercise therapy, including the effects on the metabolic health of pregnant women after childbirth, the risk of cardiovascular disease, and the long-term health of offspring, will provide more comprehensive guidance for clinical practice. In addition, the use of big data and artificial intelligence technology to integrate and analyze a large amount of exercise therapy data of GDM patients and to mine the potential rules and influencing factors of exercise therapy will also be an important direction for future research (85).

### Potential of innovative exercise therapy in gestational diabetes mellitus

Innovative exercise therapy brings new hope to the treatment of GDM. For example, the integration of virtual reality (VR) and augmented reality (AR) technologies into exercise therapy can increase the fun and attractiveness of exercise and improve exercise compliance of pregnant women by creating an immersive exercise environment. Imagining pregnant women taking virtual outdoor walks or fitness classes in VR scenes can reduce the boredom of exercise.

Additionally, personalized motion feedback systems combined with smart devices have potential applications. The system can adjust the exercise program according to the real-time exercise data and physiological indicators of pregnant women to ensure the safety and effectiveness of exercise. For example, when the exercise intensity of pregnant women is too high, the system automatically sends reminders and adjusts the follow-up exercise plan. There are also some new exercises such as yoga and Pilates, which may have a positive impact on the psychological state of pregnant women while improving their physical flexibility and balance ability and help to alleviate stress and anxiety during pregnancy. The application of these exercises in the treatment of GDM deserves further study (91).

### Multidisciplinary collaboration in exercise therapy for gestational diabetes mellitus

Exercise therapy for gestational diabetes requires a multidisciplinary collaboration. Obstetricians and gynecologists play a leading role in diagnosing GDM, assessing the physical condition of pregnant women, and formulating overall treatment plans. Sports medicine experts can formulate scientific and reasonable exercise prescriptions according to the specific conditions of pregnant women, including the type, intensity, and frequency of exercise, and can guide safety precautions during exercise. Nutritionists can provide personalized dietary advice to pregnant women, combined with exercise therapy, to control blood sugar and weight.

**Figure 5:**
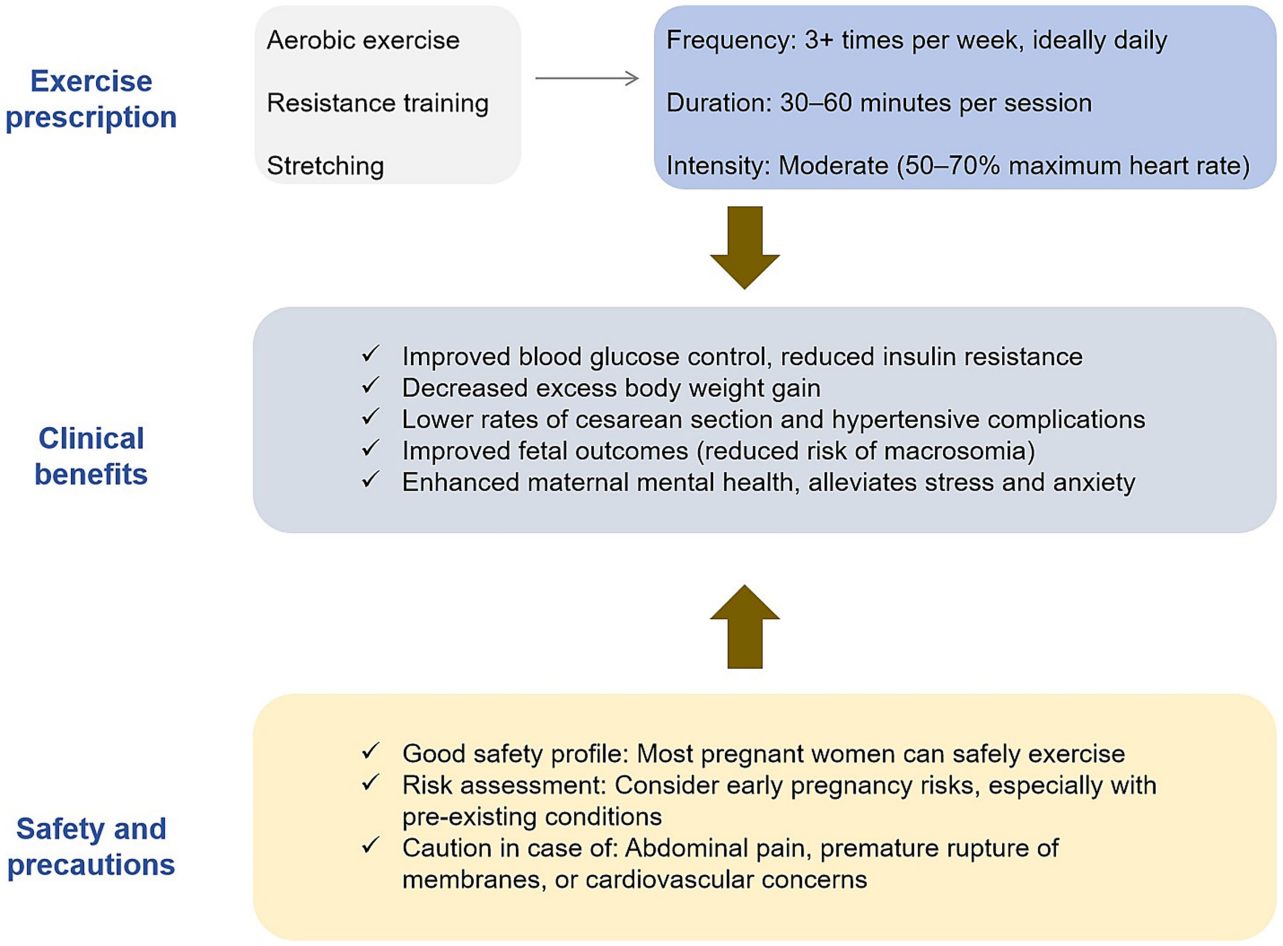
Advances in exercise therapy technology and future prospects his figure highlights the advancements in exercise therapy for gestational diabetes mellitus (GDM), including the development of exercise monitoring technologies, digital health tools, and virtual health platforms. It also presents the future prospects of personalized exercise therapy based on big data, AI, and multi-disciplinary collaboration, as well as the use of AI and machine learning to optimize exercise prescriptions for pregnant women.

In addition, psychologists and counselors can pay attention to the psychological state of pregnant women during pregnancy, help alleviate anxiety and depression caused by illness, and improve their compliance of pregnant women with exercise therapy. Multidisciplinary teams can work together to conduct research to explore more effective treatment models and interventions. For example, by studying the effects of comprehensive measures such as exercise, diet, and psychological interventions on GDM patients, we can provide a more comprehensive basis for clinical practice. This model of multidisciplinary collaboration will provide patients with gestational diabetes with better and more comprehensive medical services and improve their pregnancy outcomes and long-term health (92) (Figure 5).
